# EndoBarrier™ Implantation Rapidly Improves Insulin Sensitivity in Obese Individuals with Type 2 Diabetes Mellitus

**DOI:** 10.3390/biom11040574

**Published:** 2021-04-14

**Authors:** Anna Obermayer, Norbert J. Tripolt, Faisal Aziz, Christoph Högenauer, Felix Aberer, Florian Schreiber, Andreas Eherer, Caren Sourij, Vanessa Stadlbauer, Eva Svehlikova, Martina Brunner, Nandu Goswami, Harald Kojzar, Peter N. Pferschy, Thomas R. Pieber, Harald Sourij

**Affiliations:** 1 Division of Endocrinology and Diabetology, Medical University of Graz, 8010 Graz, Austria; a.obermayer@medunigraz.at (A.O.); norbert.tripolt@medunigraz.at (N.J.T.); faisal.aziz@stud.medunigraz.at (F.A.); felix.aberer@medunigraz.at (F.A.); eva.svehlikova@medunigraz.at (E.S.); martina.brunner@medunigraz.at (M.B.); harald.kojzar@medunigraz.at (H.K.); peter.pferschy@medunigraz.at (P.N.P.); thomas.pieber@medunigraz.at (T.R.P.); 2 Division of Gastroenterology and Hepatology, Medical University of Graz, 8010 Graz, Austria; christoph.hoegenauer@medunigraz.at (C.H.); florian.schreiber@medunigraz.at (F.S.); andreas.eherer@medunigraz.at (A.E.); Vanessa.stadlbauer@medunigraz.at (V.S.); 3 Department of Internal Medicine, Division of Cardiology, Medical University of Graz, 8010 Graz, Austria; caren.sourij@medunigraz.at; 4 CBmed—Center for Biomarker Research in Medicine, 8010 Graz, Austria; 5 CRC—Clinical Research Center, Medical University of Graz, 8010 Graz, Austria; 6 Otto Loewi Research Centre, Physiology Division, Medical University of Graz, 8010 Graz, Austria; nandu.goswami@medunigraz.at

**Keywords:** EndoBarrier™, obesity, duodenal-jejunal bypass liner, type 2 diabetes mellitus, Botnia clamp

## Abstract

The EndoBarrier™ medical device is a duodenal-jejunal bypass liner designed to mimic the effects of gastric bypass surgery to induce weight loss and glycaemic improvement. In this study, 10 participants with type 2 diabetes mellitus (T2DM), a mean body mass index (BMI) of 43.3 ± 5.0 (kg/m^2^) and a mean glycated haemoglobin A1c (HbA1c) of 60.6 ± 8.6 mmol/mol were examined at baseline (before implantation of EndoBarrier™), 4 weeks after implantation, at 36 weeks (right before explantation) and 24 weeks after the removal of the device to explore the short and long-term effects on glucose metabolism. Besides a significant reduction in body weight and fat mass, EndoBarrier™ treatment significantly improved insulin sensitivity during Botnia clamp investigations after four weeks of implantation. The beneficial effects decreased over time but remained significant 24 weeks after removal of the device.

## 1. Introduction

The global burden of overweight and obesity has become a major health challenge over recent decades. Amongst a number of detrimental health consequences, obesity in particular, significantly increases the risk for development of type 2 diabetes mellitus (T2DM). By 2045, an estimated 700 million people will suffer from overt diabetes mellitus and a further 374 million people will live with impaired glucose tolerance [1]. T2DM is associated with a reduction in life expectancy of up to 7 years in men and women [2] and has a significant negative impact on global health care budgets.

Very low calorie diets improve glucose control and can even lead to diabetes remission in people with T2DM [3]. Besides medication therapy, bariatric surgery is another efficient option to improve glucose metabolism in morbidly obese people. Moreover, bariatric interventions in obese individuals with T2DM reduce cardiovascular events as well as overall mortality [4]. However, bariatric surgery represents a largely irreversible anatomical change of the gastrointestinal tract, that bears a certain risk for surgical complications and can lead to chronic adverse events such as malabsorption syndromes [5].

The EndoBarrier™, a 60 cm long fluoropolymer sleeve, developed by GI Dynamics (Lexington, MA, USA) is intended to be used as a device to induce weight loss. It can be inserted endoscopically and the nitinol anchor secures the Endobarrier™ in the duodenal bulb. The device unfolds through the duodenum and the proximal part of the jejunum. It delays the absorption of nutrients by preventing the contact of chyme with the intestinal mucosa of the duodenum [6]. Due to the manufacturer’s recommendations, it is designed to remain in situ for 12 months. The main effects include significant weight loss and an improvement in glucose control [7]. However, the mechanisms of the improvement in glucose metabolism have not yet been thoroughly studied.

The aim of this study was to assess glycaemic effects of the EndoBarrier™ in obese participants with T2DM by performing Botnia clamp (intravenous glucose tolerance test followed by hyperinsulinaemic euglycemic clamp) and a mixed meal tolerance test before implantation, 4 weeks after the implantation of the EndoBarrier™, at 36 weeks when it was removed, as well as 24 weeks after the removal of the device.

## 2. Materials and Methods

### 2.1. Study Design

This was an open, prospective, single-center, single-arm pilot study, serving as a basis for an adequately powered trial with the Endobarrier™ in people with diabetes and/or non-alcoholic fatty liver disease in the future. Ten obese participants with a BMI between 30.0 and 49.0 kg/m^2^ and established T2DM with suboptimal glycaemic control (HbA1c ≥ 6.5% (48 mmol/mol)) were enrolled in this study. Inclusion, exclusion criteria and study procedures are described in detail in the study protocol [8]. The study was approved by the local ethical committee of the Medical University of Graz (EC number 26–280 ex 13/14) and the participants were recruited from the outpatient clinic at the Division of Endocrinology and Diabetology at the Department of Internal Medicine, Medical University Graz, Austria.

### 2.2. Endobarrier™ Device

The device was implanted and explanted under general anesthesia by trained gastroenterologists. Participants were advised to take omeprazole 40 mg twice daily starting 3 days before the implantation until 2 weeks after explantation of the EndoBarrier™ device (GI Dynamics, Lexington, MA, USA). Participants were instructed to follow a liquid diet for 2 weeks after the implantation and change slowly to a normal diet with no macronutrient restrictions over the following 10 days. Biopsies of the upper gastro-intestinal tract were taken prior to implantation and after explantation of the EndoBarrier™.

### 2.3. Examinations

For trial purposes, participants underwent physical examinations, blood sampling, Botnia clamps, mixed meal tolerance tests, lactulose/mannitol tests and dual-energy X-ray absorptiometry (DXA; GE Healthcare, Waukesha, WI, USA) measurements including body composition before the implantation, 4 and 36 weeks after the implantation, as well as 24 weeks after the explantation. Body composition was assessed using DXA to determine lean mass, fat mass, bone mineral content and total body composition. A self-administered, semi-quantitative FFQ (Food Frequency Questionnaire) was used at every visit to assess usual food consumption.

Changes in cardiovascular risk factors were calculated by the UKPDS (UK Prospective Diabetes Study) risk engine.

Routine parameters were determined using a Cobas analyzer (Roche Diagnostics, Mannheim, Germany).

Blood samples for glucagon like peptide 1 (GLP-1) were collected in pre-chilled tubes containing EDTA + aprotinin. After centrifugation, plasma samples were frozen at − 80 °C until analysis. For the determination of human glucagon like peptide 1 (GLP-1) a commercially available ELISA kit was used (active GLP-1 ELISA (GLP-1 (7–36) and (9–36), ALPCO Diagnostics, Salem, NH, USA)). The test was performed according the instructions provided by the distributor [8].

### 2.4. Statistical Analysis

As we performed a pilot trial involving Botnia clamps, we did not perform a formal sample size estimation. However, post hoc power analysis showed, that our study had more than 90% power to demonstrate the observed difference.

All data are presented as mean ± standard deviation. Data were checked for distribution normality by the Shapiro–Wilk test. The Friedman test was applied to compare parameters over time and the Durbin–Conover test with Bonferroni corrections was applied for post hoc multiple pairwise comparisons of parameters. The weight adjusted glucose infusion rate (GIR) was calculated with a multilevel non-linear mixed model with post hoc multiple comparison and Bonferroni correction. All statistical analyses were performed using SPSS 22 software for Windows (SPSS Inc., Chicago, IL, USA, 2018). A *p*-value of <0.05 was considered statistically significant.

## 3. Results

All of the 10 enrolled participants (6 female) completed the study. The mean age was 48 ± 9 years, duration of T2DM was 7 ± 6 years.

Mean body weight decreased from 121.2 ± 18.5 kg to 116.3 ± 18.2 kg (*p* = 0.006) after 4 weeks of EndoBarrier™ therapy and to 115.1 ± 21.4 kg (*p* = 0.075 vs. baseline) until explantation of the device after 36 weeks. However, there was a slight increased to 117.2 ± 20.8 kg (*p* = 0.117 vs. baseline) 24 weeks after explantation.

Baseline fat mass measured by DXA was 58.1 ± 12.0 kg and decreased to 55.0 ± 12.5 kg after 4 weeks (*p* = 0.006) and to 53.6 ± 15.2 kg after 36 weeks (*p* = 0.021) but increased again 24 weeks after explantation to 54.3 ± 15.2 kg and was not significantly different from baseline (*p* = 0.141). No changes in gut permeability were observed. Detailed results are presented in Table 1.

### 3.1. GIR

**Figure 1:**
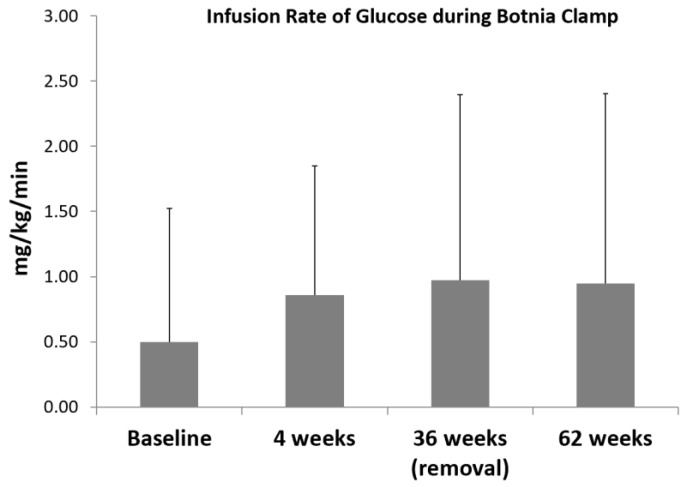
Infusion rate of glucose during 4 Botnia clamp investigations; data are mean ± SD, Baseline vs. 4 weeks *p* = 0.038; Baseline vs. 36 weeks; *p* = 0.001; Baseline vs. 24 weeks after explantation *p* = 0.001.

The mean glucose infusion rate (GIR) during Botnia clamps adjusted for weight increased from 0.50 ± 0.60 mg/kg/min at baseline to 0.86 ± 0.72 mg/kg/min after 4 weeks (*p* = 0.038) indicating a higher insulin sensitivity. A total of 36 weeks after insertion of the EndoBarrier™ device, the mean GIR remained significantly higher than at baseline (0.97 ± 1.36 mg/kg/min, *p* = 0.001) and remained significantly increased 24 weeks after explantation with 0.95 ± 1.34 mg/kg/min (*p* = 0.001) (Figure 1).

### 3.2. Diabetes Medication

36 weeks after the insertion of the device, glucose lowering treatment was reduced in five participants, one participant remained on the same treatment and four had an intensification as compared to the baseline.

### 3.3. FFQ

**Figure 2:**
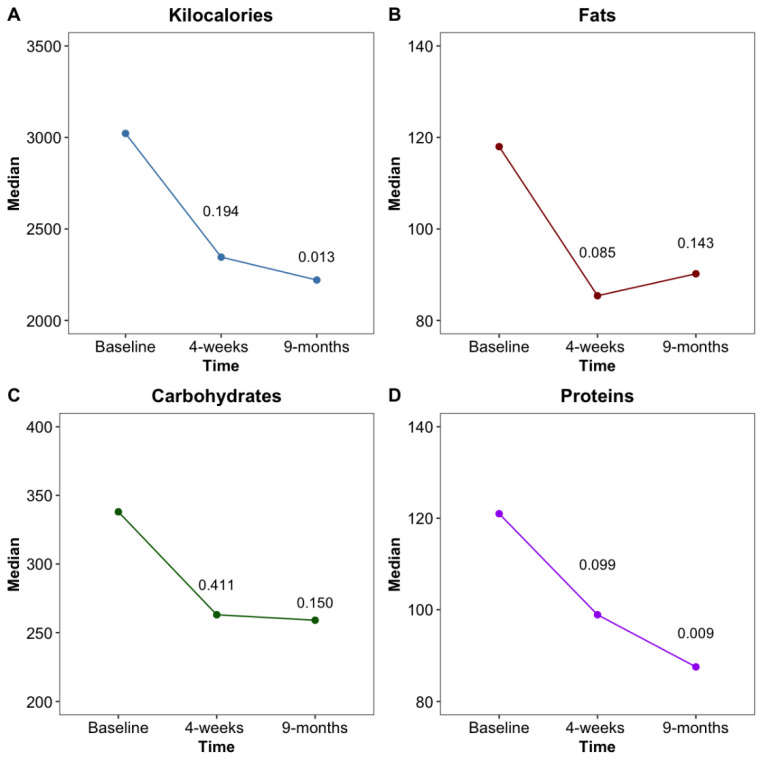
Changes of total kcal (**A**), as well as fat (**B**), carbohydrate (**C**) and protein (**D**) intake in grams at baseline, 4 weeks and 36 weeks after EndoBarrier™ implantation.

The FFQ showed a decrease in kcal (kilocalorie) consumed after the implantation of the EndoBarrier™ with a significant reduction (*p* = 0.013) after 36 weeks. While fat and carbohydrate intake reduced numerically over the time period, according to the FFQ, both did not meet the statistical significance level. However, protein consumption was significantly decreased at 36 weeks compared to the baseline (*p* = 0.009) (Figure 2).

No changes in plasma protein levels were observed throughout the study (7.25 (IQR 6.85–7.65) mg/dL at baseline versus 7.1 (IQR 6.9–7.3) mg/dL at 9 months, *p* = 0.763).

### 3.4. Biopsies

Biopsies were performed before implantation and at explantation of the EndoBarrier™ to investigate atrophic effects of the device. No signs of villous atrophy were observed in the 10 study participants.

### 3.5. Adverse Events

During the study period, four serious adverse events in four participants were reported. These resulted from one case of dehydration, one case of duodenal ulcer which was treated with sucralfate, in additionally to a high dose proton pump inhibitor, one case of prolonged nausea and vomiting requiring intravenous fluid replacement and one case of haemorrhoid bleeding. Participants experiencing an adverse event did not lose more weight as compared to those without adverse events. No premature removal of the EndoBarrier™ was required.

## 4. Discussion and Conclusions

We observed significant changes in body fat, accompanied by rapid improvements in insulin sensitivity assessed by clamp technique in obese people with T2DM treated with the EndoBarrier™ device.

While a number of studies [7,9,10] have reported significant improvements in glucose parameters observed with the duodenal-jejunal bypass liner, reaching a maximum at 36 weeks after implantation, we were able to demonstrate a significant and sustained effect of the device on insulin sensitivity already at 4 weeks after the implantation of the device. HbA1c was reduced but this did not reach statistical significance. This could be due to adjustments in the glucose lowering medication made due to improvements in glucose levels to prevent hypoglycemia.

A significant reduction in total calories as well as a significant reduction in proteins was observed at 36 weeks after implantation. Plasma protein levels were checked at every visit and no significant change in plasma proteins was observed in our study.

Our data suggest, that the EndoBarrier™ not only reduces the contact of the chyme with the intestinal mucosa, but it also leads to reduced calorie intake. This is most likely due to the bloating or the nausea that occurs if people continue eating the usual amount of food while having the device implanted.

While we observed a partial regain in body weight and increase in HbA1c 24 weeks after the explantation of the EndoBarrier™ device, which is in line with the recent findings of Deutsch et al. [11], insulin sensitivity remained sustainably improved even 24 weeks after the explanation of the device as compared to baseline.

Previous research showed, that the Endobarrier™ device was more effective than calorie restriction only [12], however, was less effective than bariatric surgery, an intervention that can lead to a weight reduction of more than 30% [13]. The Endobarrier™ can be placed endoscopically and provides a minimally invasive, reversible option which does not change the anatomy of the digestive tract permanently as is the case with bariatric surgery. It can be used for high-risk patients as a pre-bariatric surgical intervention to lose weight before the surgery [10]. However, the Endobarrier™ device causes gastrointestinal side effects, which occurred in 40% of our participants. Similar figures have been observed in previous trials [14]. No device had to be removed prematurely in our study.

The lactulose/mannitol ratio as a measure of gut permeability remained unchanged by the implantation of the device.

While in our study no cases of hepatic abscesses occurred, within the ENDO trial (Safety and Efficacy of EndoBarrier in Subjects With Type 2 Diabetes Who Are Obese, NCT01728116), the number of abscesses in people receiving the Endobarrier™ device was higher than expected (3.5%), leading to the FDA halting the trial in March 2015 [15]. The remaining uncertainty around the causes for these adverse events led to postponement of our previously planned larger, multicenter study in people with diabetes and/or non-alcoholic fatty liver disease after the pilot phase.

We believe that the Endobarrier™ device can provide a potential temporary, reversible and minimally invasive option in people with diabetes and severe insulin resistance as well as people with non-alcoholic fatty liver disease who need to lose weight. As the device seems to be less effective than bariatric surgery, it might represent an important intermediate step between conventional medical and surgical therapy.

In conclusion, in this study we observed a rapid improvement in insulin sensitivity four weeks after the implantation of the EndoBarrier™ device assessed by Botnia clamps, an effect that was sustained and still persistent after explantation of the device.

## Figures and Tables

**Table 1:** Comparison of outcome parameters from baseline to 4 and 36 weeks after implantation and 24 weeks after removal of the EndoBarrier™; data are presented as mean ± SD.

	Baseline	4 Weeks	36 Weeks after Implantation	24 Weeks after Explantation	*p*-Value1	*p*-Value2	*p*-Value3
Body weight (kg)	121.2 ± 18.5	116.3 ± 18	115.1 ± 21.4	117.2 ± 20.8	0.006	0.075	0.117
Body Mass Index (kg/m^2^)	43.3 ± 5.0	41.2 ± 4.8	40.6 ± 5.8	41.4 ± 6.0	0.006	0.075	0.117
Fat mass (kg)	58.1 ± 12	55.0 ± 12.5	53.6 ± 15.2	54.3 ± 15.2	0.021	0.021	0.141
C-peptide/Glucose Ratio	0.020 ± 0.015	0.019 ± 0.009	0.022 ± 0.012	0.018 ± 0.009	0.420	1.000	1.000
HbA1c (mmol/mol)	60.6 ± 8.6	57.4 ± 8.6	55.1 ± 11.7	66.1 ± 21.2	1.000	0.414	1.000
Glucose (mg/dL) AUC	440 ± 61	402 ± 107	458 ± 110	580 ± 170	0.819	1.000	0.576
C-peptide (ng/mL) AUC	11.6 ± 6.7	10.7 ± 6.3	12.5 ± 7.8	12.0 ± 3.8	1.000	1.000	0.465
Early insulin response	−0.02 ± 4.91	−1.00 ± 11.72	9.48 ± 23.50	0.63 ± 0.49	0.741	1.000	1.000
Fasting glucose (MMTT)	153 ± 28	160 ± 82	155 ± 51	170 ± 42	1.000	1.000	0.654
QUICKI	0.267 ± 0.026	0.283 ± 0.029	0.277 ± 0.038	0.290 ± 0.022	0.225	0.339	0.279
Lactulose/Mannitol Ratio	0.011 ± 0.010	0.011 ± 0.10	0.039 ± 0.072	0.005 ± 0.008	1.000	0.114	0.214
ALT	33 ± 17	32 ± 13	28 ± 10	31 ± 9	1.000	0.375	1.000
AST	26 ± 7	31 ± 14	25 ± 6	35 ± 11	0.492	1.000	0.132
GGT	49 ± 40	53 ± 60	35 ± 26	65 ± 83	1.000	0.120	1.000
UKPDS CHD	14.1 ± 17.9	14.3 ± 18.7	16.3 ± 20.4	13.6 ± 10.6	1.000	1.000	1.000
UKPDS Fatal CHD	8.4 ± 11.6	8.6 ± 12.4	9.3 ± 13.1	8.6 ± 8.6	1.000	1.000	1.000
GLP-1 (pmol/L)	27.9 ± 13.3	24.2 ± 9.2	18.2 ± 11.4	36.1 ± 56.8	1.000	0.081	1.000

AUC: Area under the Curve; ALT: Alanine transaminase; AST: Aspartate aminotransferase; GGT: Gamma-glutamyl transferase; *p*-value1: Comparison Baseline vs. 4 Weeks; *p*-value2: Comparison Baseline vs. 36 weeks; *p*-value3: Comparison Baseline vs. 24 weeks after explantation. UKPDS CHD: UK Prospective Diabetes Study coronary heart disease, GLP-1: Glucagon-like peptide-1, MMTT: mixed meal tolerance test, GIR: glucose infusion rate.

## Data Availability

The dataset generated and analysed in this study is not publicably available but may be obtained from the corresponding author upon a reasonable request.
